# Ketogenic Diet: A Dietary Modification as an Anxiolytic Approach?

**DOI:** 10.3390/nu12123822

**Published:** 2020-12-14

**Authors:** Adam Włodarczyk, Wiesław Jerzy Cubała, Aleksandra Wielewicka

**Affiliations:** Department of Psychiatry, Faculty of Medicine, Medical University of Gdańsk, 80-952 Gdańsk, Poland; cubala@gumed.edu.pl (W.J.C.); wielewicka.aleksandra@gmail.com (A.W.)

**Keywords:** GABA, ketogenic diet, low-carbohydrate, anxiety, ketosis, gut microbiota, nutritional psychiatry, mental health, nutrition

## Abstract

Anxiety disorders comprise persistent, disabling conditions that are distributed across the globe, and are associated with the high medical and socioeconomic burden of the disease. Within the array of biopsychosocial treatment modalities—including monoaminergic antidepressants, benzodiazepines, and CBT—there is an unmet need for the effective treatment of anxiety disorders resulting in full remission and recovery. Nutritional intervention may be hypothesized as a promising treatment strategy; in particular, it facilitates relapse prevention. Low-carbohydrate high-fat diets (LCHF) may provide a rewarding outcome for some anxiety disorders; more research is needed before this regimen can be recommended to patients on a daily basis, but the evidence mentioned in this paper should encourage researchers and clinicians to consider LCHF as a piece of advice somewhere between psychotherapy and pharmacology, or as an add-on to those two.

## 1. Introduction

Anxiety disorders comprise a group of persistent, disabling conditions that are distributed across the globe, and are associated with a high burden of the disease being a great cost in the course of healthcare expenses, due to commonly ruling one out from social, professional, and/or educational duties [1,2,3,4,5,6,7].

Although intensive research on genetics, neuroimaging, blood-testing, and neurochemical markers has been carried out, the studies failed to determine the anxiety biomarkers, as the majority of them showed solitary findings which sometimes were neither replicable, nor consistent with each other [8].

The array of treatment modalities is still limited in efficacy with regard to remission, prognosis, and relapse prevention. There is an unmet need for novel strategies in the treatment of anxiety disorders, including treatments that fall outside of pharmacotherapy and psychosocial intervention.

### 1.1. Neurotransmission and Gut-Microbiota Interplay in Anxiety

#### 1.1.1. Monoamines

Within the exploration of the possible biological causes of anxiety, there is evidence on serotonergic and noradrenergic transmission defects in the mechanism of anxiety; there is a need to explore more treatment options to treat these disorders, and a diet regimen could be one of them. The monoaminergic hypothesis led to the development of selective and nonselective inhibitors of serotonin transporters and/or norepinephrine, with the aim of monoaminergic transmission augmentation [9,10,11]. There is a strong correlation between enhanced noradrenergic activity and fear and anxiety. Additionally, the neurons of the chief noradrenergic projection center in the central nervous system, the locus coeruleus, are hyperactive during anxiety, and the excitation of this part of the brain is related to symptoms such as stress and anxiety responses [9]. Furthermore, pharmacotherapeutic confirmation points towards the involvement of the serotonergic system in the brain [9].

#### 1.1.2. Hypothalamic-Pituitary-Adrenal Axis, Divalent Ions, Inflammation, and Reactive Oxygen Species in Anxiety

Another factor is the disturbance of the hypothalamic–pituitary–adrenal axis (HPA), which is seen to have elevated cortisol levels. However, hypocortisolism has also been noted [12]. Factors such as the divalent ions of zinc or magnesium (the digestion of which is controlled partly by the human microbiota [13]) may exert effects on the progression of the cortical brain-derived neurotrophic factor (zinc and magnesium), *N*-methyl-d-aspartate (NMDA) antagonists’ mechanisms of action, and neuromodulation. The mechanism of action also highlights the linkage between anxiety and some divalent ion deficiencies [14,15]. Inflammation and oxidative stress are also being linked to the anxiety process. The former was consistently found to affect anxiety-related brain regions, i.e., the anterior cingulate cortex, amygdala, and insula, which may result from cytokine effects on monoamines and glutamate. Increased inflammatory cytokines are, in turn, associated with increased oxidative stress, and the generation of reactive oxygen species (ROS) and reactive nitrogen species. The latter could be linked with obsessive-compulsive disorder and panic disorders’ etiologies, which show statistically-significant levels of some antioxidant enzymes and malondialdehyde [16,17,18].

#### 1.1.3. Excess Glutamate

With regard to glutamate, its relationship to anxiety has also already been established. This chief excitatory neurotransmitter in the human brain was found to play a vital role in anxiety. The mechanism consists of NMDA receptor complex activation, which requires both glutamate (which could be depleted by the LCHF diet, as described below) and its co-agonist, glycine. d-cycloserine, for instance, being a partial agonist—at the glycine recognition site—of the glutamatergic NMDA receptor, can act as a cognitive enhancer to augment exposure strategies during the cognitive-behavioral therapy of anxiety disorders [19,20].

#### 1.1.4. GABA Deficiency

Additionally, the main inhibitory gamma-aminobutyric acid (GABA) dysfunctions have been discussed in studies as being responsible for mood fluctuations in affective disorders and the psychopathology of fear (the acquisition, storage, and extinction of fear memory); this has not only been proven theoretically but also practically, by the rapid reduction of symptomatology, anxiety, and sleep disorders when allosteric modulators of GABA were given [21,22,23]. In patients with General Anxiety Disorder (GAD), the number of GABA type A (GABA-A) receptors is reduced in the temporal lobe [24]; patients with panic disorders also have reduced GABA-A receptor numbers in the parietal, temporal and frontal cortexes, the left hippocampus, and the precuneus [25]. Likewise, GABA is responsible for the inhibition of cortisol excretion in stress, and corticotropin-releasing hormone excretion, which also supports the hypothesis that, when altered, GABA could intensify the risk of depression and/or anxiety [23]. Persistent Selective Serotonin Reuptake Inhibitor (SSRIs) intake enhances the cortical GABA concentrations observed in both patients and healthy controls, and are compatible with the antidepressant drug-induced potentiation of GABA release as a mechanism underlying antidepressant effects. Similarly to SSRIs, tricyclic antidepressants that increase the concentration of noradrenaline and serotonin take part in GABAergic transmission modulation. The noradrenergic neuration of GABAergic interneurons increases the GABAergic transmission in the frontal, sensorimotor, and entorhinal cortices; parts of the hippocampus; and the basolateral amygdala. Additionally, significant decreases in the left temporal pole GABA-A receptors were found in a PET study with female GAD patients. Studies have shown that infusions of GABA or GABA-A receptor agonists into the amygdala lessened the measures of anxiety in several animal subjects, while infusions of GABA antagonists managed to show anxiogenic properties [26]. The role of GABA has long been observed as being central to the regulation of anxiety, and this neurotransmitter system is the target of benzodiazepines (and related drugs) used to treat anxiety disorders effectively [27].

#### 1.1.5. Gut Microbiota

Furthermore, the intestinal microbiota have various functions in the organism, including the synthesis of certain bacteria groups that replenish the absorption of ions, calcium and iron, and the transformation of fatty acids, stimulating the development of the immune system and protective functions [13]. The relationship between the development of depression, the immune response, and bowel function is currently explained by the phenomenon of ‘leaky gut syndrome’. The research revealed that ‘tight junctions’—connections between the cells of the intestinal epithelium—deteriorate under stress, which in turn leads to the translocation of intestinal bacteria through the intestinal barrier into the circulatory system [28].

To summarize, at present, antidepressants augment monoaminergic transmission and also strengthen GABA transmission, the lowered concentration of which is frequently observed in anxiety disorders [9,23].

Among monoaminergic drugs, cognitive-behavioral therapy, or occasional benzodiazepine use, there is a lot to be discovered in the nutrition regimen regarding decreasing anxiety symptoms. The aim of this mini-review is to bring together the existing knowledge of the ways in which certain types of food components affect anxiety.

### 1.2. Low Carbohydrate Diets and Their Hypothesized Impact on Anxiety Treatment

#### 1.2.1. Low-Carbohydrate Diets

Dietary modification as a treatment intervention modality has been widely discussed since the 19th century [29,30,31]. A very low-carb diet (up to ca. 50 g carbohydrates per day [32]), the LCHF-ketogenic diet (KD), was the typical treatment for diabetes mellitus (DM) throughout the 19th century [33,34]. A dietary regimen that provided ketosis was found in the treatment amended by the physicians of ancient Greece, including for epilepsy, by altering their patients’ diet, mostly by the ‘complete abstinence of food and drink’ [35].

Diets with low amounts of carbohydrate consumption (low-carb) seem promising both for weight mass optimization among mentally ill patients and for their possible anxiolytic effect. A diet is characterised as being low-carb high-fat (LCHF) when fat comprises >70% of the daily calorie consumption, with sugars being 5–15%, and the rest of the calories being supported by proteins [32].

Although there are various types of LCHF diets, like the Atkins diet, modified Atkins diet, low-glycemic index treatment diet, and the medium-chain triglyceride (MCT) KD [36,37,38], we will focus on the biological aspect of the mechanism of ketosis. As has previously been said, a very low-carb KD and starvation have something in common, and the process is called ketosis. The difference between physiological ketosis and pathological ketoacidosis (which is seen in DM type 1 or prolonged starvation) is a major limiting factor in the production of ketones [39]. Ketosis, the state of the overproduction of acetoacetate, d-3-hydroxybutyrate, and acetone (called collectively ‘the ketone bodies’) by the liver, takes place when carbohydrates are removed from the diet (or during starvation). Ketosis seems to only ‘imitate’ starvation, being different from it, as the daily caloric intake stays on a normal, or even higher, level. The restriction of carbohydrates to under 50 g induces glycogen depletion and ketone production due to the mobilization of fat stored in the adipose tissue, which is the main mechanism associated with a decrease in body weight. Very low-carbohydrate diets and mild low-carbohydrate diets (the latter is commonly defined as carbohydrate consumption up to 130 g per day) differ in the type of body mass loss. In the review by Hashimoto et al. 2016, very low-carb diets were associated with a decrease in fat mass, but mild low-carb diets were not associated with a decrease in fat mass, although both were associated with bodyweight decrease [40]. Furthermore, the Prospective Urban Rural Epidemiology (PURE) study [41] showed that high carbohydrate consumption (over 60% of daily calories) was linked with an adverse impact on total mortality and non-cardiovascular disease mortality. On the other hand, higher fat consumption was associated with a lower risk of total mortality, non-cardiovascular disease mortality, and stroke [41].

The direct and indirect influence on the central nervous system of KD can be observed in the increasing of the cerebral blood flow, and the decreasing the mammalian target of rapamycin (mTOR) [42] by the increase of the level of endothelial nitric oxide synthase protein expression, but also passively (indirectly). The indirect, ‘passive’, effects on the central nervous system are supposed to be mediated by microbiota through an increase of short-chain fatty acids and a decrease of GABA [43]. Bacteria such as *Akkermansia muciniphila* and *Lactobacillus* are known as short-chain fatty acid producers [44]. It is known that the KD induces anorexigenic effects: decreased adenosine monophosphate-activated protein (AMP) phosphorylation, and an increase of post-meal free fatty acids. KD has also appetite stimulant (orexigenic) abilities: it increases the brain’s GABA concentrations of AMP, and decreases reactive oxygen species (ROS) [45]. 

In a study on the KD mechanism in epilepsy treatment—by Calderon et al.—in which rodents were set on a two weeks KD trial, the ketone levels in their urine were measured along with GABA, glutamate levels, and weight. Not only did the rats on KD gain weight by only about 1.2 g, whilst the control group gained 20.8 g, but the levels of their neurotransmitters changed significantly in favor of GABA. In probes of microdialysate, the glutamate levels declined non-significantly between KD (3.5 ± 0.6 μM) and the control group (5.18 ± 0.73 μM) (*p* = 0.08), while the GABA levels were significantly higher (47 ± 8 nM) in rats kept in the KD group compared to the control rats (26 ± 3 nM) (*p* ≤ 0.03) [45]. This mechanism of KD could be supportive of anxiety disorder treatments. 

#### 1.2.2. Gut Microbiota and the Steroid Pathway in the Potentiation of GABA Transmission in Low-Carbohydrate Diets

Furthermore, GABA can be synthesized by the gut microbiota residents: Lactobacilli and Bifidobacteria (Lactobacillus brevis, Bifidobacterium dentium, Bifidobacterium adolescentis, and Bifidobacterium infantis). Lactobacillus rhamnosus has been proven for its therapeutical potential in modulating the expression of central GABA receptors, mediating depression and anxiety-like behaviors [46], which links the possible anxiolytic outcome effect with the gut microbiome. It was suggested that the LCHF diet, and—in general—the inhibition of glycolysis in the brain, could reduce neuronal excitability through the potentiation of GABA transmission via the steroid pathway [47,48]. Forte et al. [47] reported a novel mechanism for the reduction of network hyperexcitability by the inhibition of glycolysis, which involves the potentiation of the shunting inhibition in excitatory neurons, in which a glucose analogue—2-deoxy-d-glucose—potentiates the extra-synaptic tonic GABAergic current through the activation of neurosteroidogenesis. There seems to exist a linkage with the gut–brain axis, neurosteroids, and GABA-A interplay, while neuronal GABA-A receptors are one of the prime molecular targets of neurosteroids [49]. As some gut microbiota residents could be called ‘manufacturers of GABA’, the gut microbiota diversity seems to influence positively the circulating steroid levels, in particular, that of allopregnanolone. Prebiotic consumption could improve frequently co-existing anxiety disorder symptoms through the promotion of undisturbed non-rapid eye movement (NREM) sleep and stress-related REM sleep rebound, and the prevention of stress-induced reductions in gut microbial alpha diversity [49,50].

Increasingly, low-carb diets are being used to treat behavioral and mood disorders such as attention deficit disorder, for which diets that are low in sugar and high in fatty acids are recommended [51]. Still, little is known about KD and gut microbiota dependence with regard to mental health. Mostly, the evidence found focuses on the effect of KD on the gut microbiota of children with either epilepsy [43,52,53,54] or autism [55]. Only some articles focus on adult patients, but most focus on subjects with significant comorbidities; such literature is to be found on sclerosis multiplex, in which KD restores the impaired gut microbiome in patients with sclerosis multiplex [56]. Similar data can be found on professional athletes; in a study by Murtaza et al. [57], the researchers found statistically significant differences in some bacteria species between the stool microbiota profiles of those athletes consuming the LCHF diet compared with their baseline measurements. Moreover, tests performed on mice suggest a beneficial role of KD for gut microbiota [43,58]. As a ketogenic diet modifies the gut microbiome, the preservation of proper gut health through the implementation of fermented food (i.e., yogurt, water and milk kefir, kimchi, fermented vegetables) or pre/probiotics consumption (which does not interfere with the assumptions of KD) seem important. It is possible that taking probiotics could help prevent composition disorders of the gut microbiota as a consequence of chronic stress, and the depletion of inflammation and the increasing of serotonin biosynthesis probiotics could be an element of anxiety disorder relapse prevention. 

#### 1.2.3. Anti-Inflammatory Effect of the Ketogenic Diet and Fatty Acids

It is hypothesized that a ketogenic diet may reduce inflammation [59]. Compared with glucose metabolism, the metabolism of ketone bodies produces fewer ROS, which contribute to inflammation. Ketolytic metabolism produces fewer free radicals and ROS, affecting the mitochondrial Q coenzyme pair and the cytoplasmic glutathione couple [59,60].

**Figure 1:**
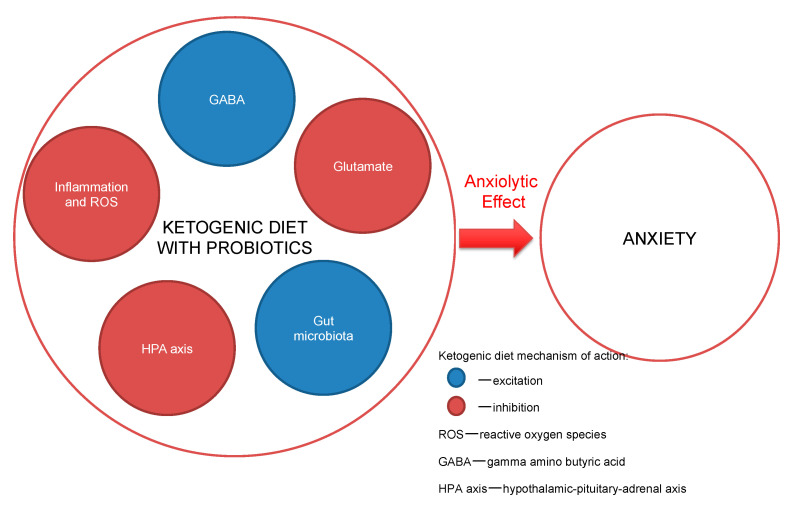
Ketogenic diet mechanisms of action.

Some research indicates the benefit in the outcome of anxiety when the consumption of particular fats in the diet is increased, i.e., the essential polyunsaturated fatty acids (EPUFAs), also called vitamin F, and omega-3 fatty acids. The clinician-advised dosing of the two omega-3 fatty acids—eicosapentaenoic acid (EPA) and docosahexaenoic acid (DHA)—is at least 1.5–2.5 g daily consumption [61,62]. The American Psychiatric Association guidelines support omega-3 consumption for the mentally ill, through the consumption of at least 1 g of EPA and DHA daily, which is in-line with the guidelines of the American Heart Association [63]. DHA plays a role in the brain’s cellular structure construction, because as much as 20% of the brain is composed of it. All omega-3 formulations exhibit anti-inflammatory activity and help to maintain brain cells’ stability, with linkages to neurotransmitters’ (serotonin, dopamine) proper functioning [64]. Nowadays, with higher depression morbidity in society, studies are showing that omega-3 fatty acids are eaten rarely and in lower doses than in the past decades [64,65]. The proposed mechanisms of action are presented in Figure 1.

#### 1.2.4. The ‘Ketogenic Menu’

The ‘clean keto’ version of KD is mostly based on ‘healthy’ macronutrients, such as low-processed food, i.e., fat sources such as free-range egg yolks, and polyunsaturated fatty acids such as olive, canola, and grapeseed oil, oily fish, and nuts. As for proteins, fish, meat, cheese, egg whites (mostly high-fat protein sources) are recommended, and carbohydrates are limited to mostly unprocessed, low glycemic index carbohydrates (which are ‘smuggled’ through green vegetable consumption, brown rice, etc.). A professional dietician’s guidance is advised. The previously-mentioned divalent ions linked to anxiety can also be supplied in the LCHF menu, i.e., through zinc-rich foods such as oysters (which are low-carbohydrate meals) and other seafood, etc. Magnesium can be found mostly in green leafy vegetables, while selenium is found in seafood, poultry, fish, and eggs, which all are favorable choices in LCHF diets [35]. 

A study on over 121,000 participants concluded that high stress and high neuroticism levels were associated with poorer diet quality; however, poor diet quality did not predict emotional or mental health problems [66]. Although the data suggest that enhancing diet quality may not hold promise in preventing mental disorders, patients may benefit from a specific type of nutrition regimen whilst they are mentally ill [67,68]. These findings could help establish the right dietary regimen to enhance the GABAergic transmission and support the gut–brain axis.

## 2. Conclusions

Although there is a growing body of literature that links nutrition to mood, little can be found on the proposed biological mechanisms of action of certain micro- and macronutrients on neurotransmission, leaving studies with, mainly, epidemiological data [69,70]. There is also evidence with regard to the gut–brain axis, in which some species of bacteria have the ability to generate the neuroendocrine hormones and/or neuroactive compounds involved in a key aspect of neurotransmission [46], which may be responsible for the anxiolytic effect. There is also the vital fact that metabolic acidosis—which is a potentially life-threatening condition that can appear due to ketoacidosis caused by starvation, diabetes, lactate acidosis, alcohol ingestion, or renal failure—is also represented by ketone bodies in the urine and blood, but it differs in those levels of in the blood and urine (due to the lower blood pH in ketoacidosis than in physiological ketosis) [71].

The findings rationalize the need for more detailed, longitudinal research on the ways in which diet and microbiome interactions may be better understood and managed in order to optimize the reduction of anxiety for the benefit of the patients. LCHF diets, in some anxiety disorders, may provide a rewarding outcome, but more research is needed before this regimen can be recommended to patients on a daily basis; however, the evidence mentioned in this paper should encourage psychiatrists to recommend LCHF diets as advice somewhere between psychotherapy and pharmacology, or as an add-on to those two. In our mind, the LCHF diet is a promising, well-accepted diet regimen which has an impact on anxiety disorders, supporting mainly long-term relapse prevention strategies, in combination with the already-approved strategies.
